# A Novel Function for KLF4 in Modulating the De-Differentiation of EpCAM^−^/CD133^−^ nonStem Cells into EpCAM^+^/CD133^+^ Liver Cancer Stem Cells in HCC Cell Line HuH7

**DOI:** 10.3390/cells9051198

**Published:** 2020-05-12

**Authors:** Zeynep Firtina Karagonlar, Soheil Akbari, Mustafa Karabicici, Eren Sahin, Sanem Tercan Avci, Nevin Ersoy, Kıvılcım Eren Ates, Tugsan Balli, Bilge Karacicek, Kubra Nur Kaplan, Canan Celiker, Nese Atabey, Esra Erdal

**Affiliations:** 1 Genetics and Bioengineering Department, Izmir University of Economics, Izmir 35330, Turkey; zeynep.firtina@ieu.edu.tr; 2 Department of Medical Biology and Genetics, Faculty of Medicine, Dokuz Eylul University, Izmir 35340, Turkey; soheil.akbari@ibg.edu.tr (S.A.); sahineren87@gmail.com (E.S.); sanemtercan@gmail.com (S.T.A.); 3 Izmir Biomedicine and Genome Center, Izmir 35340, Turkey; mustafa.karabicici@ibg.edu.tr (M.K.); nevinersoy@gmail.com (N.E.); bilge.karacicek@ibg.edu.tr (B.K.); kaplan.kub@gmail.com (K.N.K.); clkrcnn@gmail.com (C.C.); nese.atabey@ibg.edu.tr (N.A.); 4 Department of Histology and Embryology, Faculty of Medicine, Dokuz Eylul University, Izmir 35340, Turkey; 5 Department of Pathology, Faculty of Medicine, Cukurova University, Adana 01260, Turkey; kivilcimerenates@hotmail.com; 6 Department of Radiology, Faculty of Medicine, Cukurova University, Adana 01260, Turkey; tugsanballi@gmail.com

**Keywords:** hepatocellular carcinoma (HCC), liver cancer stem cells, tumor plasticity, KLF4, reprogramming, EpCAM

## Abstract

The complex and heterogeneous nature of hepatocellular carcinoma (HCC) hampers the identification of effective therapeutic strategies. Cancer stem cells (CSCs) represent a fraction of cells within tumors with the ability to self-renew and differentiate, and thus significantly contribute to the formation and maintenance of heterogeneous tumor mass. Increasing evidence indicates high plasticity in tumor cells, suggesting that non-CSCs could acquire stem cell properties through de-differentiation or reprogramming processes. In this paper, we reveal KLF4 as a transcription factor that can induce a CSC-like phenotype in non-CSCs through upregulating the EpCAM and E-CAD expression. Our studies indicated that KLF4 could directly bind to the promoter of *EpCAM* and increase the number of EpCAM^+^/CD133^+^ liver cancer stem cells (LCSCs) in the HuH7 HCC cell line. When KLF4 was overexpressed in EpCAM^−^/CD133^−^ non-stem cells, the expressions of hepatic stem/progenitor cell genes such as *CK19*, *EpCAM* and *LGR5* were significantly increased. KLF4 overexpressing non-stem cells exhibited greater cell viability upon sorafenib treatment, while the cell migration and invasion capabilities of these cells were suppressed. Importantly, we detected an increased membranous expression and colocalization of β-CAT, E-CAD and EpCAM in the KLF4-overexpressing EpCAM^−^/CD133^−^ non-stem cells, suggesting that this complex might be required for the cancer stem cell phenotype. Moreover, our in vivo xenograft studies demonstrated that with a KLF4 overexpression, EpCAM^−^/CD133^−^ non-stem cells attained an in vivo tumor forming ability comparable to EpCAM^+^/CD133^+^ LCSCs, and the tumor specimens from KLF4-overexpressing xenografts had increased levels of both the KLF4 and EpCAM proteins. Additionally, we identified a correlation between the KLF4 and EpCAM protein expressions in human HCC tissues independent of the tumor stage and differentiation status. Collectively, our data suggest a novel function for KLF4 in modulating the de-differentiation of tumor cells and the induction of EpCAM^+^/CD133^+^ LCSCs in HuH7 HCC cells.

## 1. Introduction

Hepatocellular carcinoma (HCC) is the most common type of primary liver cancer and the fourth leading cause of cancer-related deaths worldwide [1,2]. Inter- and intra-tumor heterogeneity is a major hallmark of HCC and significantly contributes to treatment failure and chemotherapeutic resistance in HCC patients [3,4,5,6].

The cancer stem cell theory, which supports the existence of cells within the tumor possessing stem cell-like properties, provides an explanation for the extensive phenotypic and functional heterogeneity in tumors [7,8,9,10,11]. Cancer stem cells (CSCs) or tumor-initiating cells represent a group of cells within the tumor with the ability to self-renew and differentiate, and thus could significantly contribute to the production of heterogeneous differentiated tumor mass [7,8,9,10,11]. A growing number of studies indicate that CSCs are responsible for HCC metastasis, recurrence after resection and therapy resistance of advanced HCC cells [12,13,14,15,16]. The origins of CSCs and their molecular mechanism in regards to cancer relapse and resistance are still not well-defined; however, in the recently proposed CSCs plasticity model, tumor cells are believed to represent a very plastic and dynamic population, with the ability to continuously shift between non-CSC and CSC states [17,18,19,20]. The ability to describe the type of intrinsic or extrinsic stimuli that modulate this shift and the transcription factors that regulate the CSCs phenotype are of great importance in targeting these cells in HCC therapy [21]. 

EpCAM, one of the first markers identified to isolate putative liver cancer stem cells (LCSCs), is normally expressed by hepatoblasts/human hepatic progenitor cells in the liver, but it is not expressed by mature hepatocytes [22,23,24,25]. Yamashita and his colleagues showed that EpCAM-positive HCC displayed a unique molecular signature with features of hepatic progenitor cells and expressed known stem/progenitor markers, such as CK19 and c-Kit, while EpCAM-negative HCC displayed genes associated with features of mature hepatocytes [26,27]. Another cell surface marker CD133/Prominin-1 has also been identified as a biomarker for CSC in various cancers, including HCC [28,29]. Moreover, cells expressing both the EpCAM and CD133 surface markers were shown to possess more characteristics of LCSCs in HuH7 cells and exhibit a higher tumor initiating ability [30]. Notably, higher EpCAM and/or CD133 levels were shown to predict poor survival in various cancers, such as colon, prostate, lung and breast cancer [30,31,32,33,34,35,36]. Despite its importance, the regulation of the EpCAM^+^/CD133^+^ cell population and its clinical significance in HCC still remains largely unknown.

KLF4, a well-known Yamanaka factor, is a complex transcription factor that, depending on the context, can act as a transcriptional activator, a transcriptional repressor, an oncogene or a tumor suppressor [37,38,39]. Although there are conflicting studies about the role of KLF4 in HCC tumorigenesis, KLF4 is repeatedly reported by recent studies as a regulator of CSCs in various cancers [40,41,42,43,44]. However, the role of KLF4 in the regulation and maintenance of an LCSC population is yet to be determined. In this paper, we show that KLF4 is a transcription factor that drives a stemness phenotype in the HuH7 HCC cell line by upregulating the EpCAM and E-CAD expression. Our results identify KLF4 as one of the transcription factors that can modulate the de-differentiation of tumor cells and regulate the shift between non-CSC and CSC states in HuH7 cells. 

## 2. Materials and Methods

### 2.1. Isolation of LCSCs and Non-stem Cells from HuH-7

The Huh7 cell line, originally from Jack Wands Laboratory at Massachusetts General Hospital, Boston, MA, was a gift from Dr. Mehmet Ozturk, IBG, Turkey. The authentication of the HuH7 cell line was achieved by DNA profiling at the University of Colorado Cancer Center (UCCC) DNA Sequencing & Analysis Core (CO, USA) using Applied Biosystem’s Identifiler kit (PN4322288). In order to isolate the LCSCs (EpCAM^+^/CD133^+^) and non-stem cells (EpCAM^−^/CD133^−^) from the HuH7 cell line, cells were stained with EpCAM-FITC (#130-110-998, Miltenyi Biotech, Auburn, AL, USA) and CD133-APC (#130-111-000, Miltenyi Biotech, Auburn, AL, USA) antibodies, and sorted using BD FACS Aria III cell sorter. Cells were cultured as previously described [45].

### 2.2. Overexpression of KLF4 in EpCAM-/CD133- Non-stem Cells

The lentiviral expression construct of mCherry-tagged *KLF4* (pLM-mCherry-*KLF4*) was a gift from Michel Sadelain (Addgene plasmid # 23243) [46], and the mock vector was generated by removing the *KLF4* gene with Age-I and Sal-I enzymes from this plasmid. Lentiviral particles were produced in HEK293T cells using the trans-lentiviral ORF packaging kit (#TLP5919, Dharmacon). After 12–16 h of infection, the HEK293T cell medium was replaced with reduced serum-DMEM. The following day, viral particles were collected, filtered and added onto the EpCAM-/CD133- cells with 8 mg/mL polybrene (#H9268-5G, Merck). The virus was removed after 16 h, and the cells were incubated with a fresh medium for 2 additional days before use in the experiments.

### 2.3. RT-qPCR

Total RNA was isolated using the GeneJET RNA purification kit (#K0732, Thermo Fisher) and the RNA concentration was detected using NanoDrop (Thermo Fisher Scientific). One microgram of RNA was then converted to cDNA using a Maxima First Strand cDNA Synthesis Scientific kit (#K1642, Thermo Fisher Scientific). For the real-time quantitative RT-PCR, expression levels were determined in quadruplicate on a 7500 Fast RT PCR System (Applied Biosystems), using the TaqMan Universal Master Mix (#4304437, Thermo Fisher Scientific). The relative gene expression was normalized to the *RPL41* gene and calculated by using the 2^−ΔΔCt^ method. 

### 2.4. Chromatin Immunoprecipitation Assay (ChIP) and ChIP-qPCR

The chromatin immunoprecipitation assay (ChIP) was performed using EZ-Magna ChIP A/G (#17-10086, Merck) according to manufacturer’s instructions. The DNA protein complexes in the lysates were subjected to immunoprecipitation using antibodies anti-KLF4 (#ab106629, Abcam) or the control normal IgG (which EZ-Magna kit includes). The isolated DNA was used as a template in the PCR with specific oligonucleotides flanking the *EpCAM* promoter regions containing the putative KLF4-binding sites that were obtained from the JASPAR database and previous studies (C/AC/AACA/GCCCT/A and G/AG/AGG C/TGC/T) [47]. The primers were chosen from the most representative sites for the putative KLF4 binding sites lying between 2000 bp upstream and 100 bp downstream of the transcription start site (TSS) in the *EpCAM* promoter region (*EpCAM* >chr2:47594287-47596386). ChIP-PCR reactions with the promoter region-specific primers were performed using the “Fisher Applied Biosystems/Fast7500” system and TaqMan Universal Master Mix II (#4304437, Thermo Fisher Scientific). The amplification reaction was carried out for 40 cycles (95 °C 15 s, 60 °C 45 s) after denaturation at 95 °C for 15 min. The Ct values were determined for each primer after the amplification and the fold change in the amount of DNA was calculated and normalized according to the negative control IgG.

### 2.5. Immunofluorescence Staining

After the cell sorting, LCSCs (EpCAM^+^/CD133^+^) and non-stem cells (EpCAM^−^/CD133^−^) were seeded in 24-well plates as 35 × 10^3^ cells/well. The next day, the cells were fixed with 4% PFA, rinsed with 1X PBS and then permeabilized using a 0.5% TritonX (#28313, Thermo Fisher Scientific). After the cells were incubated with a blocking buffer for 2 h at room temperature, staining was carried out using the following primary antibodies: EpCAM (VU1D9)-Alexa Fluor488 Conjugate (#cs5198, Cell signaling), E-CAD (#sc8426, Santa Cruz) and β-CAT (D10A8) (#cs8480, Cell Signaling). For the F-actin staining, the Phalloidin-iFlour 555 reagent (#ab176756, Abcam) was used. Cells were visualized using a confocal microscope (# LSM880, Zeiss).

### 2.6. Colony Forming and Spheroid Formation Assays

For the spheroid formation assay, 1 × 10^3^ sorted LCSCs (EpCAM^+^/CD133^+^) and 1 × 10^3^ sorted non-stem cells (EpCAM^−^/CD133^−^) were seeded in 24-well ultra-low attachment plates in DMEM/F12 (#31330-038, Gibco) supplemented with 2% B27 (#12587010, Gibco), 1.8% BSA (#A9418, Merck), EGF (20 ng/mL) (#af-100-15, Peprotech), bFGF (10 ng/mL) (#100-18B, Peprotech), insulin (5 μg/mL) (#I3146, SigmaAldrich) and heparin (# H3149, 50 ng/mL) [30]. On the fifth day, spheroids were counted under a microscope. All experiments were performed in triplicate and repeated three times.

For the colony forming assay, 2 × 10^2^ sorted cells were seeded into a well of a six-well plate. After 12 days, the cells were washed twice with precooled PBS and fixed with 4% paraformaldehyde (PFA) (#158127, Merck) for 5 min at room temperature (RT). The fixed cells were then stained with 0.1% crystal violet for 5 to 10 min, and a count was made of the number of colonies, defined as >50 cells/colony.

### 2.7. MTT Assay

Cells were grown in 96-well plates (1 × 10^3^ cells per well) for 2 d, then treated with 2 μM sorafenib for 72 h. After 72 h, cell viability was assessed using the 3-(4,5-dimethylthiazol-2-yl)-2,5-diphenyltetrazolium bromide (MTT) reagent (#CT01-5, Merck) according to the manufacturer’s instructions as previously described [48]. 

### 2.8. Migration and Invasion Assay

In vitro motility and invasion assays were performed as previously described [48]. Briefly, 2 × 10^4^ cells were seeded into the upper chamber of a 24-well transwell plate (Corning) in a 100 µL reduced serum (2%) medium. A 10% FBS-containing culture medium was added to the lower chamber, and the cells were incubated at 37 °C for 24 h. After 24 h, the upper cells were removed with a cotton swab, while the lower cells, which had migrated into the lower chamber, were fixed and stained using the Diff Quick kit (Siemens Healthcare Diagnostics). Cells that had migrated were counted for each chamber using a bright field inverted microscope. Measurements were performed for at least three technical and two biological replicates. Fold differences were calculated by dividing the experimental results by the control.

### 2.9. Transfection and Luciferase Reporter Assay

Cells were seeded into 24-well plates. After the overnight incubation, the cells were transfected with a TOP-FLASH firefly luciferase vector (M50) and pRL-TK plasmid vector using the FuGENE HD (#E2311, Promega) transfection reagent. After transfection, the cells were treated with 80 ng/mL Wnt3a. The luciferase activity was measured by the Promega Dual Luciferase Reporter Assay kit (#E1910, Promega) according to manufacturer’s instructions. The levels of the reporter gene induction in transiently transfected cells were normalized to the Renilla luciferase enzyme activity. 

### 2.10. Xenotransplantation (In vivo Tumorigenicity Assay)

All animal experiments were conducted in accordance with the Institutional Animal Use and Care Committee. For the tumorigenicity assay, 25 × 10^3^ mock (EpCAM^−^/CD133^−^ ), 25 × 10^3^ overexpressing cells (EpCAM^−^/CD133^−^ KLF4 OE cells) and 25 × 10^3^ EpCAM+/CD133+ cells were injected subcutaneously into 6–8-week-old NSG (NOD-scid IL2Rgamma^null^) mice (n = 7 for each group). Tumor growth was monitored every two days after the 10th day of injection. The experiments were terminated at day 30. The tumors were extracted, fixed in 10% formalin and embedded in paraffin. Four-micrometer-thick sections of the paraffin-embedded tissue blocks were used for the immunohistochemical (IHC) studies. Tumor sections were dewaxed in xylene and rehydrated in an alcohol series in a decreasing concentration. The sections were heated by a microwave for 20 min in a citrate buffer (pH 6.0) for the antigen retrieval, and then were incubated with 3% hydrogen peroxide for blocking the endogenous peroxidase activity. After the incubation in the blocking serum for 2 h, the samples were stained with EpCAM (#ab32392, Abcam) and KLF4 (#ab215036, Abcam) antibodies overnight at 4 °C. The next day, after washing with PBS three times, sections were incubated with biotinylated secondary antibodies, and then with streptavidin conjugated horseradish peroxidase for 15 min according to kit instructions (#85-9043, Invitrogen). The sections were finally stained with DAB (diaminobenzidine) (#1718096, Roche), and then counterstained with Mayer hematoxylin and evaluated under a light microscope.

### 2.11. Ethics Statement

Human tissues were collected with the required approval from the Institutional Review Board (Cukurova University Medical School, Adana, Turkey) and patient’s written consent, which conformed to the ethical guidelines of the 1975 Helsinki Declaration. Fifty patients (Supplementary Table S1) were included in the study. In the analysis, the following were taken into consideration: tumor size, tumor number, tumor type, presence of vascular invasion, metastasis and serum alpha-fetoprotein (AFP) level, in addition to patient age and sex. Resected surgical specimens were subjected to IHC to identify the protein expression of EpCAM, and KLF4. Tumors were graded using the criteria proposed by Edmondson and Steiner: (I) Well-differentiated; (II) moderately differentiated; (III) poorly differentiated; and (IV) undifferentiated.

### 2.12. IHC Staining

The HCC specimens collected from patients who had undergone resection were fixed in 10% formalin and embedded in paraffin. Four-micrometer-thick sections of the paraffin-embedded tissue blocks were used for the IHC studies. After the tumor specimens were stained with EpCAM (#ab32392, Abcam) and KLF4 (#ab215036, Abcam) antibodies, the following criteria were used for the assessment of the protein expression level: 0 (negative, <5%); 1+ (weak, 5%–30%); 2+ (moderate, 30%–60%); and 3+ (strong, >60%). The evaluation of the tissue staining was performed independently by two experienced pathologists. All specimens were scored blind and a final grade was calculated as the average of the two independent grades.

### 2.13. Statistical Analysis

A statistical analysis was carried out using GraphPad Prism (GraphPad Software, Inc., San Diego, CA, USA). Statistical methods included ANOVA and Student’s *t*-test. Differences between groups were considered significant at * *P* < 0.05, ** *P* < 0.001 and *** *P* < 0.0001. 

A statistical analysis was carried out using GraphPad Prism (GraphPad Software, Inc., San Diego, CA, USA). Statistical methods included ANOVA and Student’s *t*-test. Differences between groups were considered significant at * *P* < 0.05, ** *P* < 0.001 and *** *P* < 0.0001. 

## 3. Results

### 3.1. Characterization of Liver Cancer Stem Cells (LCSCs) and Non-Stem Cells in HuH-7

**Figure 1:**
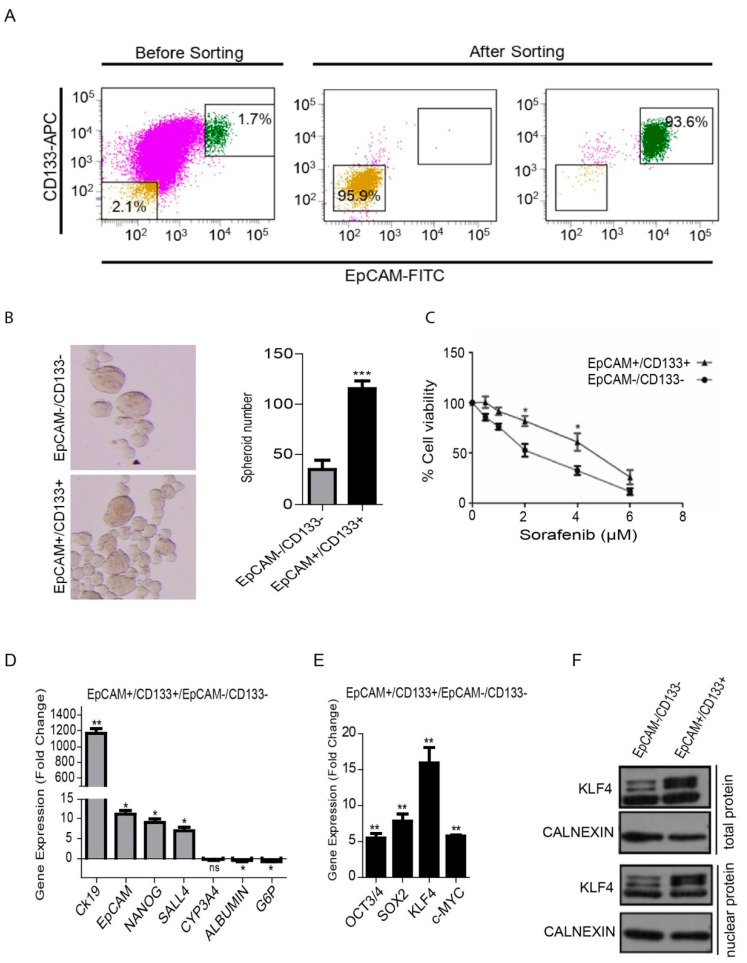
Isolation and analysis of the EpCAM^+^/CD133^+^ liver cancer stem cell subpopulation from the HuH-7 cell line. (**A**) Analysis of the EpCAM and CD133 surface marker expressions in the HuH-7 cell line by flow cytometry. The HuH7 cell line was stained with EpCAM-FITC and CD133-APC antibodies, and was sorted using a BD FACS Aria III Cell Sorter. The sorted EpCAM^+^/CD133^+^ and EpCAM^−^/CD133^−^ cells were stained after cell sorting to determine purity. (**B**) The spheroid formation ability of cells was tested in ultralow attachment plates. Representative images of the spheres formed are shown. Column graphs demonstrate the number of spheres calculated at the end of the experiment. (**C**) LCSCs and non-stem cells were treated with sorafenib (0 μM–8 μM) and the viability curve was calculated by a MTT test. (**D**) Stem/progenitor and hepatocyte marker gene expression and (**E**) the gene expression of the reprogramming factors in LCSCs and non-stem cells were analyzed using real-time PCR. Column graphs show the fold change normalized to RPL41, and calculated by using the 2^−ΔΔCt^ method. (**F**) Western blotting analysis demonstrated a higher KLF4 expression in the whole lysate and nuclear protein extracts of EpCAM^+^/CD133^+^ LCSCs. Data represent the average of at least three independent experiments. * *p* < 0.05, ** *p* < 0.001, *** *p* < 0.0001. Error bars indicate standard deviation (SD).

The flow cytometry analysis of the HuH-7 parental cell line showed that the cancer stem cell population carrying the EpCAM and CD133 surface markers (EpCAM^+^/CD133^+^) constituted around 1%–10% of the parental population. For our experiments, the EpCAM^+^/CD133^+^ LCSCs and EpCAM^−^/CD133^−^ non-stem cells were sorted out from the parental cell line (Figure 1A). After sorting, the EpCAM^+^/CD133^+^ LCSCs and EpCAM^−^/CD133^−^ non-stem cells were maintained in a cell culture only up to day 5 before use in the experiments, since they have a tendency to lose their phenotype in a prolonged cell culture (Supplementary Figure S1). When we compared the stemness-related properties of the two cell populations, EpCAM^+^/CD133^+^ LCSCs showed an increased spheroid formation ability (Figure 1B) and exhibited a greater cell viability upon sorafenib treatment (Figure 1C). There is evidence that cancer stem cells exhibit gene expression patterns in common with early embryonic stem cells [8,9]. As expected, EpCAM^+^/CD133^+^ LCSCs exhibited a higher expression of several stem cell-associated genes such as CK19, EpCAM, NANOG and SALL4, while mature hepatocyte-associated genes such as ALB and G6P were significantly lower in these cells compared with EpCAM^−^/CD133^−^ non-stem cells (Figure 1D). Moreover, EpCAM^+^/CD133^+^ LCSCs expressed higher levels of the four transcription factors, OCT3/4, SOX2, KLF4 and c-MYC, often referred to as Yamanaka factors, which are capable of reprogramming terminally differentiated adult cells into an induced pluripotent state (Figure 1E). Among these factors, KLF4 had the highest expression level in EpCAM^+^/CD133^+^ LCSCs. The Western blot analysis showed that the KLF4 protein can be detected both in the whole lysate and the nuclear protein extracts of EpCAM^+^/CD133^+^ LCSCs (Figure 1F).

### 3.2. KLF4 Increases Stemness Phenotype via Transcriptional Regulation of EpCAM

**Figure 2:**
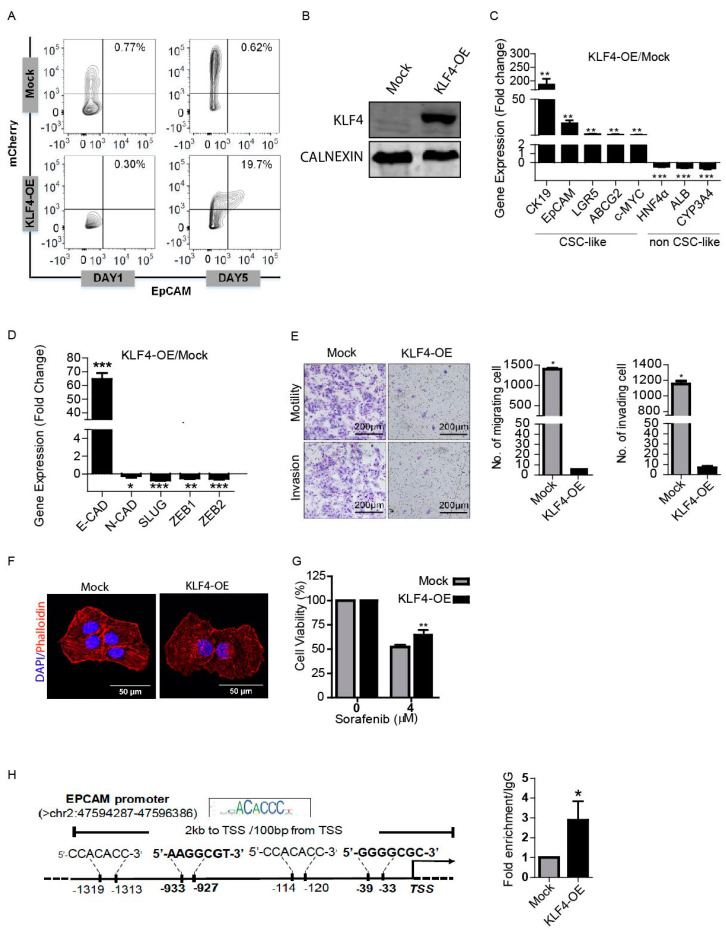
KLF4 modulates stemness and the phenotypic shift of non-stem cells. (**A**) Non-stem cells were transduced with KLF4-mCherry and mock mCherry plasmids. The effect of KLF4 on the EpCAM cell population was analyzed by flow cytometry. (**B**) Western blotting analysis of KLF4 expression in the mock vs. the overexpressing cells. (**C**) Transcriptional expression of cancer stem cell (CSC)- and non-CSC-related genes were measured by real-time PCR. Column graphs show the fold change normalized to RPL41 and calculated by using the 2^−ΔΔCt^ method. (**D**) Epithelial and mesenchymal marker gene expressions were analyzed using real-time PCR. Column graphs show the fold change normalized to RPL41 and calculated by using the 2^−ΔΔCt^ method. (**E**) Non-stem cells were transduced with KLF4-mCherry and mock-mCherry plasmids and then the migration and invasion capacity of the cells were assessed by the transwell chambers. Representative images of migrated and invaded cells are given. The number of stained cells is presented as column graphs on the right. (**F**) KLF4-overexpressing and mock cells were stained with tetramethylrhodamine (TRITC)-labeled phalloidin (red) to demonstrate F-Actin filament organization. Cells were counterstained with DAPI (blue) to show the nuclei. Images were captured by confocal microscopy. (**G**) Cell viability of KLF4-overexpressing and mock cells against sorafenib was measured by MTT assay. (**H**) Binding of KLF4 on the EpCAM promoter was analyzed using chromatin immunoprecipitation (ChIP) assay followed by real-time PCR. Data represent the average of at least three independent experiments. * *p* < 0.05, ** *p* < 0.001, *** *p* < 0.0001. Error bars indicate SD.

We initially tested whether the introduction of Yamanaka factors (OSKM) could induce EpCAM^+^/CD133^+^ LCSCs in the HuH7 cell line. Overexpression of all four Yamanaka factors (OSKM) together induced EpCAM^+^/CD133^+^ LCSC population in the non-stem cells (Supplementary Figure S2), however, importantly, when they were applied individually, only KLF4 was able to induce the EpCAM^+^/CD133^+^ population (Figure 2A,B). Thus, in this study, we worked on dissecting the individual role of KLF4 in the induction of EpCAM^+^/CD133^+^ LCSC, and the effect of this induction on the tumorigenicity of HuH7 cells. When we analyzed the differentiation-related genes in KLF4-overexpressing non-stem cells, the expression of hepatic cancer stem cell-like genes, including CK19, EpCAM and LGR5, were significantly increased, while the expression of hepatocyte-like genes, including HNF4a, ALB and CYP3A4, were significantly decreased in these cells, suggesting that the KLF4-overexpressing cells obtained a less differentiated phenotype (Figure 2C). Furthermore, we analyzed the effect of a KLF4-induced phenotypic shift to the stemness state on the sequential changes in epithelial and mesenchymal markers. Consistent with previous studies, KLF4-overexpressing cells had significantly lower expression levels of the mesenchymal genes, including N-CAD, SLUG, ZEB1 and ZEB2, while the E-CAD expression in these cells was greatly increased (> 60-fold) (Figure 2D). Accordingly, the migration and invasion capacities of the KLF4-overexpressing cells were significantly lower compared with the mock transfected cells (Figure 2E), with the KLF4-overexpressing cells exhibiting a more epithelial cell-like actin cytoskeleton organization (Figure 2F). Moreover, the KLF4-overexpressing cells exhibited greater cell viability upon sorafenib treatment compared with the mock plasmid transfected cells, confirming the drug resistance of the KLF4-induced stem-like cells (Figure 2G).

To determine a direct regulatory role of KLF4 on the EpCAM expression, we performed a chromatin immunoprecipitation (ChIP) assay, followed by qPCR. Primers flanking the predicted KLF4 binding sites on the EpCAM promoter obtained from the JASPAR database and previous studies [47] (C/AC/AACA/GCCCT/A and G/AG/AGG C/TGC/T) were used to amplify the chromatin fragments enriched by KLF4 immunoprecipitation. Our results demonstrated that KLF4 recruitment on the promoter region of the EpCAM gene is significantly higher in the KLF4-overexpressing cells compared with the mock cells, suggesting that KLF4 can act as a transcriptional activator of the EpCAM gene expression (Figure 2H).

### 3.3. KLF4 Increases EpCAM, E-CAD and β-CAT Cell Membrane Expressions

**Figure 3:**
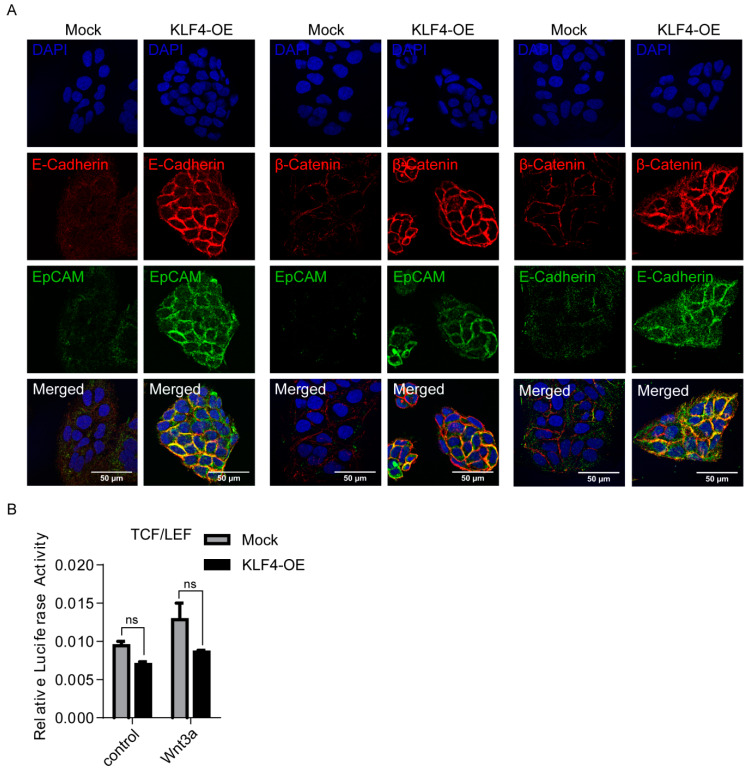
KLF4 overexpression increases EpCAM, E-CAD and β-CAT cell membrane expressions in non-stem cells. (**A**) Representative confocal images of KLF4-overexpressing and mock cells for EpCAM, β-catenin and E-cadherin expressions are shown. Nuclei were co-stained with DAPI. (**B**) SuperTOPFlash reporter and Renilla luciferase plasmids were co-transfected into KLF4-overexpressing and mock cells. Luciferase activity was measured with or without the WNT3a (80 ug/ml) recombinant protein. Data represent the average of at least three independent experiments. Error bars indicate SD.

E-CAD is a key cell adhesion protein implicated as both a tumor suppressor and promoter in human carcinomas [49,50,51]. Consistent with previous studies that identified E-CAD as a KLF4 target gene [52,53], our results also showed that the E-CAD expression is significantly increased in KLF4-overexpressing non-stem cells (Figure 2C). Although the loss of E-CAD has been correlated with poor prognosis in HCC [54,55], paradoxically, the E-CAD expression is increased in around 40% of HCCs, suggesting a requirement for E-CAD at certain stages of the tumorigenesis [56,57]. E-CAD is a binding partner of β-CAT and can promote the recruitment of β-CAT to the plasma membrane and prevent its degradation by the Axin/APC/GSK3β destruction complex. Thus, E-CAD indirectly modulates Wnt signaling by physically sequestering β-CAT at the membrane [58,59]. Recently, an E-CAD/ β-CAT complex at the membrane has been shown to promote growth factor signaling and support HCC cell survival in the initial stages of HCC progression [60]. We therefore analyzed the co-expressions of E-CAD, β-CAT and EpCAM in KLF4-overexpressing cells using confocal microscopy. Our results indicate that the expressions of EpCAM, E-CAD and β-CAT were increased, especially in the cell membranes of KLF4-overexpressing cells (Figure 3A). These results suggest that, by inducing the expression of E-CAD and EpCAM, KLF4 can promote the recruitment of β-CAT to the plasma membrane via the adherens junction (AJ) complex.

To understand the changes on the Wnt-β-CAT canonical pathway under KLF4-induced conditions, we performed luciferase assays using a β-CAT reporter plasmid containing TCF/LEF-responsive elements on both the KLF4-overexpressing and mock cells. Neither basal- nor Wnt3a-induced β-CAT transcriptional activity was altered under the KLF4 overexpression, suggesting that the KLF4-induced stemness phenotype is independent of the activation of the canonical Wnt-β catenin pathway (Figure 3B).

### 3.4. KLF4 Restores Tumorigenic Potential of Non-stem Cells

**Figure 4:**
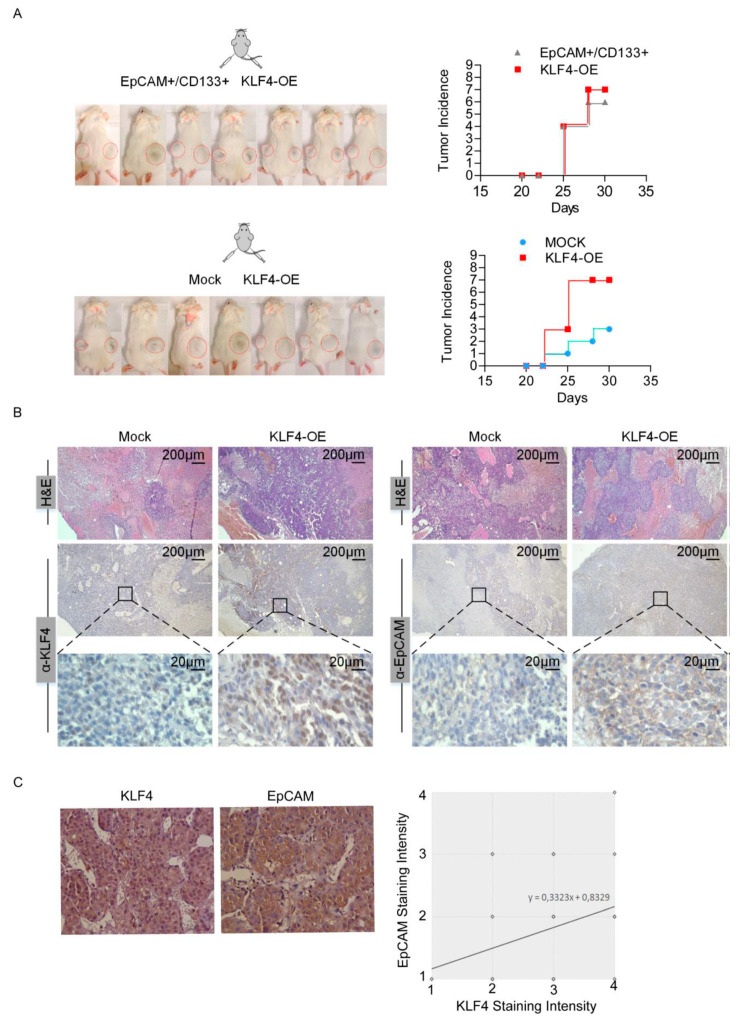
KLF4 overexpression is associated with high tumorigenic potential. (**A**) Equal number (25 × 10^3^) of LCSCs, KLF4-overexpressing non-stem cells and mock overexpressing non-stem cells were resuspended in matrigel and injected into NGS mice (n = 7 per group). The right side was injected with KLF4-overexpressing non-stem cells and the left side was injected with LCSCs (top row) or mock overexpressing non-stem cells (bottom row). Tumor development was considered to start when tumors became palpable and the incidence of palpable tumors is depicted for all experimental setups. (**B**) H and E and immunohistochemistry staining with anti-KLF4 and anti-EpCAM in sacrificed tumors. H and E: hematoxylin and eosin. (**C**) Correlation between KLF4 and the EpCAM expression was evaluated in human HCC samples. Fifty tumor biopsy samples were obtained and IHC staining was performed to detect KLF4 and EpCAM expressions. Representative images of IHC staining for EpCAM and KLF4 in human tissues are shown. EpCAM- and KLF4-positive stainings were quantified, and the correlation was analyzed with the Spearman correlation method (correlation coefficient: R = 0.33, P = 0.017).

We then tested the in vivo tumorigenicity of these cells and performed xenograft studies with the EpCAM^+^/CD133^+^ cells, mock transfected EpCAM^−^/CD133^−^ cells and EpCAM^−^/CD133^−^ cells overexpressing KLF4. While only 3 of 7 mice injected with the mock transfected EpCAM^−^/CD133^−^ cells exhibited a tumor formation, the tumor incidences were 6 out of 7, and 7 out of 7 for the mice injected with EpCAM^+^/CD133^+^ cells and mice injected with EpCAM^−^/CD133^−^ cells overexpressing KLF4, respectively, suggesting that a KLF4 overexpression restores the tumorigenic potential (Figure 4A). Furthermore, consistent with our in vitro studies, when we stained tumor sections from the mice injected with EpCAM^−^/CD133^−^ cells overexpressing KLF4, these tumor sections had increased levels of both the KLF4 and EpCAM proteins (Figure 4B). We then analyzed the KLF4 and EpCAM expressions on tumor biopsies collected from 50 HCC patients (Supplementary Table S1) and also identified a correlation between KLF4 and the EpCAM protein expression in HCC patients, independent of the tumor stage and differentiation status (Figure 4C).

## 4. Discussion

In liver cancer, as in many cancer types, phenotypic and functional tumor heterogeneity has a crucial role in tumor progression and resistance to treatment. While tumor heterogeneity can be attributed to several causes, the most important is tumor plasticity behaviour between the non-CSCs and CSCs in the tumor niche. Recent studies indicate that tumor cells may behave as a plastic and dynamic population, with the ability to shift between non-CSC and CSC states. Thus, the identification of the transcription factors that induce a cancer stem cell phenotype controlling the plasticity of tumor cell populations is of great importance for cancer therapy. 

The process of somatic reprogramming via pluripotency factors, many of which are also embryonic transcription factors that act like oncogenes, may offer an insight into how certain cells in tumor mass gain stem cell-like properties [61,62]. Notably, histologically poorly differentiated human cancers were shown to express higher levels of embryonic stem cell-like genes, supporting the possibility that these genes contribute to the reprogramming of tumor cells to a more de-differentiated stem-cell like state during tumor progression [61,62]. Similarly, there are many studies that show a higher expression of reprogramming factors in cancer stem cell populations isolated from cancer cell lines using various surface markers [63,64]. Among these factors, especially SOX2- and OCT4A-positive expressions were found to be significantly associated with a more aggressive phenotype in HCC, and both SOX2 and OCT4A were found to be independent prognostic factors for poor survival in HCC patients [65,66,67]. KLF4, which is also one of the Yamanaka factors, has essential roles in the control of self-renewal in embryonic stem cells and reprogramming of somatic cells into a pluripotent state [37,38,39]. KLF4 can function as an oncogene or tumor suppressor in a highly tissue-specific cell-dependent manner [37,38,39]. Not surprisingly, there are conflicting reports regarding KLF4 expression in HCC and its association with tumor recurrence and overall survival in HCC patients [68,69,70,71,72]. Hsu et al. demonstrated a tumor suppressive function for KLF4 in HCC [71]. Their results are in line with the results of a previous study by Li et al. [68], demonstrating that cytoplasmic KLF4 expression is significantly correlated with better tumor differentiation and favorable disease-specific survival. On the other hand, the study of Yin et al. suggests an opposite function for KLF4 in HCC [72]. Yin et al. analyzed the expression of pluripotency factors including KLF4 by real-time PCR, and found that high KLF4 expression levels were associated with aggressive tumor behaviors in terms of vascular invasion and poor tumor differentiation. High KLF4 levels were also independently associated with a poorer overall survival [72]. The discrepant results between the different studies may have arisen due to the different cohorts of samples, and different scoring criteria and techniques used for the evaluation of the immunohistochemical analysis. Moreover, the studies of Hsu et al. and Li et al. involve the evaluation of cytoplasmic KLF4. According to these authors, cytoplasmic KLF4 has a different function than nuclear KLF4, in that it possibly involves the regulation of cytoskeletal organization through interactions with an actin cytoskeleton. Thus, the KLF4 protein detected in this study, which correlated with better tumor differentiation and favorable disease-specific survival, might possibly be involved in a different function than we describe for KLF4 in our study in the de-differentiation of tumor cells into a cancer stem cell-like state. The inconsistency in and discrepancy among KLF4 reports may also reflect the pleiotropic functions of this protein and complexity in cancers, especially in hepatocarcinogenesis.

Interestingly, recent studies indicate a new role for KLF4 in various cancers involving regulating the self-renewal and maintenance of cancer stem cell populations [40,41,42,43,44]. However, the role of KLF4 in the regulation and maintenance of an LCSC population remains undetermined to date. Our data demonstrate that KLF4 could modulate the de-differentiation of HuH7 cells and induce EpCAM^+^/CD133^+^ LCSCs. We identified KLF4 as a transcriptional activator of the *EpCAM* gene, which can bind to an *EpCAM* promoter. Moreover, our data show that various stemness genes are increased in KLF4-overexpressing cells, and KLF4-induced LCSCs exhibit a gene expression signature similar to EpCAM^+^/CD133^+^ LCSCs. In various cancer cell lines, exogenous KLF4 expression is known to suppress cell motility and invasion by decreasing the mesenchymal gene expression while upregulating the epithelial gene expression [69]. Similarly, in our study, a KLF4 overexpression significantly decreased the migration and invasion of non-stem cells, while it greatly induced the expression of epithelial markers such as *CK19* and *E-CAD*. It is important to note that although these KLF4-overexpressing cells exhibited a diminished in vitro cell migration and invasion capability, their epithelial characteristics and stemness gene expression were significantly amplified. Accordingly, our in vivo xenograft studies demonstrated that with KLF4 overexpression, EpCAM^−^/CD133^−^ non-stem cells attained an in vivo tumor-forming ability comparable to EpCAM^+^/CD133^+^ LCSCs. 

E-CAD is a key cell adhesion protein and a binding partner of β-CAT, which mediates Wnt signaling by binding to the nuclear TCF/LEF transcription factors and activating the transcription of the target genes. Previous studies suggest that the loss of E-CAD causes an increase in β-CAT nuclear localization, and can induce Wnt signaling [73]. On the other hand, an increased E-CAD expression can promote the recruitment of β-CAT to the plasma membrane via the adherens junction (AJ) complex and prevent its degradation by the AXIN/APC/GSK3β destruction complex [58]. β-CAT has a different function within the AJ complex, independent of its role in canonical Wnt signaling. By acting as a bridge between α-CAT and Cadherins, β-CAT helps to maintain a proper cytoskeleton structure and to modulate cell–cell interactions and cell signaling [74]. Although an abnormal activation of the Wnt pathway plays an important role in hepatocarcinogenesis [75,76], the importance of the AJ complex-associated β-CAT in HCC initiation and progression is still unknown. Interestingly, Kim et al. recently demonstrated that membranous β-CAT interacts with multiple cadherin family members to promote the signaling of growth factor receptors, such as the epidermal growth factor receptor (EGFR), and support HCC cell survival in the early stages of HCC [60]. Since KLF4-induced LCSCs express high levels of E-CAD and EpCAM, and exhibit an epithelial stem cell phenotype, we wanted to analyze the expression and localization of β-CAT in KLF4-induced cells. Our confocal microscope analysis revealed an increased expression and colocalization of β-CAT, E-CAD and EpCAM in the KLF4-overexpressing EpCAM^−^/CD133^−^ non-stem cells. These data suggest that, by inducing the expression of EpCAM and E-CAD, KLF4 can promote the recruitment of β-CAT to the plasma membrane via the adherens junction (AJ) complex. Our studies with luciferase assays using a β-CAT reporter plasmid containing TCF/LEF-responsive elements showed that KLF4 does not affect canonical Wnt signaling. The mechanism by which KLF4 regulates stemness in HuH7 cells is probably more complex, but our results suggest a role for membranous β-CAT in the KLF4-induced stem cell phenotype in HuH7 cells, independent of its role in canonical Wnt signaling.

Our study shows a novel function for KLF4 in modulating the de-differentiation of tumor cells and the induction of EpCAM^+^/CD133^+^ LCSCs in HuH7 cells. However, our results were demonstrated only in the HuH7 cell line, a major limitation of this study. Reprogramming factors (OSKM) can be delivered to a variety of cancer cells to generate what are known as “induced pluripotent cancer cells” (iPCCs) [77,78,79]. Such iPCCs appear to have a CSC-like state after the reprogramming process [79,80,81,82]. Using a variety of HCC cells, Kim et al. demonstrated that the generation of iPCCs by a retroviral transfer of the four Yamanaka factors is possible. However, the Hep3B cell line with the null p53 gene showed better efficiency of reprogramming than the other liver cancer cell lines, suggesting that the p53 status of the cell line is important in the reprogramming efficiency [83]. It is known that the inhibition of p53 enhances the reprogramming of fibroblasts into induced pluripotent stem cells (iPSCs) [84], and that p53 inhibition can also generate CSCs from differentiated cells [85]. Thus, in order to delineate a possible regulatory role of KLF4 in the control of LCSCs, it is important to analyze the effect of KLF4 in a series of HCC cell lines with different genetic/epigenetic backgrounds. However, we must underline that a KLF4-induced state is probably a partial reprogramming, leading to the de-differentiation of HuH7 cells, rather than a full reprogramming into an iPSC state. Our results suggest that this de-differentiation is accompanied by an increase in stemness and tumorigenicity. We also believe that this KLF4-induced state is a plastic and dynamic shift of the subpopulations, and stemness will probably be lost upon continual passage. Importantly, our study is unable to provide the underlying mechanisms driving stemness features upon an increased KLF4 expression. The dual and opposing roles of KLF4 in tumorigenesis suggest that KLF4 has a complex network of protein interactions, and further studies are required to reveal these interactions. It is likely that the different effects of KLF4 on tumorigenesis depend on unidentified basal- and/or treatment-induced cellular factors and interactions. The knowledge on the role of KLF4 is expanding, and our understanding would be enhanced by the further characterization of this protein, providing more insights into its respective roles in various physiological and pathological conditions. 

## 5. Conclusions

In summary, our study demonstrates the plasticity of LCSCs in the HuH7 cell line, and identifies KLF4 as a critical inducer of this plasticity. The overexpression of KLF4 induced a partial reprogramming, leading to the de-differentiation of the HuH7 cells, which was accompanied by an increase in stemness and tumorigenicity. To determine whether KLF4 has a regulatory role in cancer stem cell populations residing in HCC tumors is yet to be tested. Nevertheless, our increasing understanding and control of tumor plasticity and de-differentiation should facilitate the exploitation of this novel concept and its application in clinical HCC treatments, which may represent a promising therapeutic strategy in various cancers.

## Supplementary Materials

The following is available online at https://www.mdpi.com/2073-4409/9/5/1198/s1, Table S1: Clinical features of HCC patients. Figure S1: Evaluation of EpCAM+/CD133+ and EpCAM-/CD133- cell populations in cell culture over days by flow cytometry. Figure S2: Overexpression of OSKM increases EpCAM+/CD133+ cell population in the HuH7 cell line.
