# Supplementary material for: A Novel Function for KLF4 in Modulating the De-Differentiation of EpCAM−/CD133− nonStem Cells into EpCAM+/CD133+ Liver Cancer Stem Cells in HCC Cell Line HuH7
Source: Cells. 2020 May 12;9(5):1198. doi: 10.3390/cells9051198 (PMC7290717; doi:10.3390/cells9051198)
Supplement: Supplementary file 1 [file cells-09-01198-s001.zip › Supplementary Table-1-06-03-2020.docx]

| **Characteristics** | **Value** |
| --- | --- |
| **Total Cases, n** | 50 |
| **Gender, n (%)** |  |
| Male | 40 (80) |
| Female | 10 (20) |
| **Age (years)** |  |
| Mean ± standart deviation | 66 ± 9,72 |
| Median (range) | 66 (38-86) |
| **Etiology, n (%)** |  |
| HBV | 38 (76) |
| Other | 12 (24) |
| **Tumor size, n (%)** |  |
| <3 cm | 3 (6) |
| 3-5cm | 13 (26) |
| >5 cm | 34 (68) |
| **Tumor type, n (%)** |  |
| Nodular | 28 (56) |
| Infitrative | 21 (42) |
| **Number of tumors, n (%)** |  |
| Single | 37 (74) |
| Multiple | 13 (26) |
| **AFP, n (%)** |  |
| <20 ng/ml | 21 (42) |
| 20-400 ng/ml | 10 (20) |
| 400-1000 ng/ml | 1 (2) |
| >1000 ng/ml | 17 (34) |
| **Fibrous Capsule, n (%)** |  |
| Absent | 33 (66) |
| Present | 16 (32) |
| **Pathological data, n (%)** |  |
| Tumor Necrosis | 16 (32) |
| Portal vein invasion | 25 (50) |
| Intrahepatic metastasis | 20 (40) |
| **Edmonson grade, n (%)** |  |
| I | 24 (48) |
| II | 18 (36) |
| III | 8 (16) |

**Table 1: Clinical features of HCC patients**
