# Supplementary figures and images for: A Novel Function for KLF4 in Modulating the De-Differentiation of EpCAM−/CD133− nonStem Cells into EpCAM+/CD133+ Liver Cancer Stem Cells in HCC Cell Line HuH7
Source: Cells. 2020 May 12;9(5):1198. doi: 10.3390/cells9051198 (PMC7290717; doi:10.3390/cells9051198)

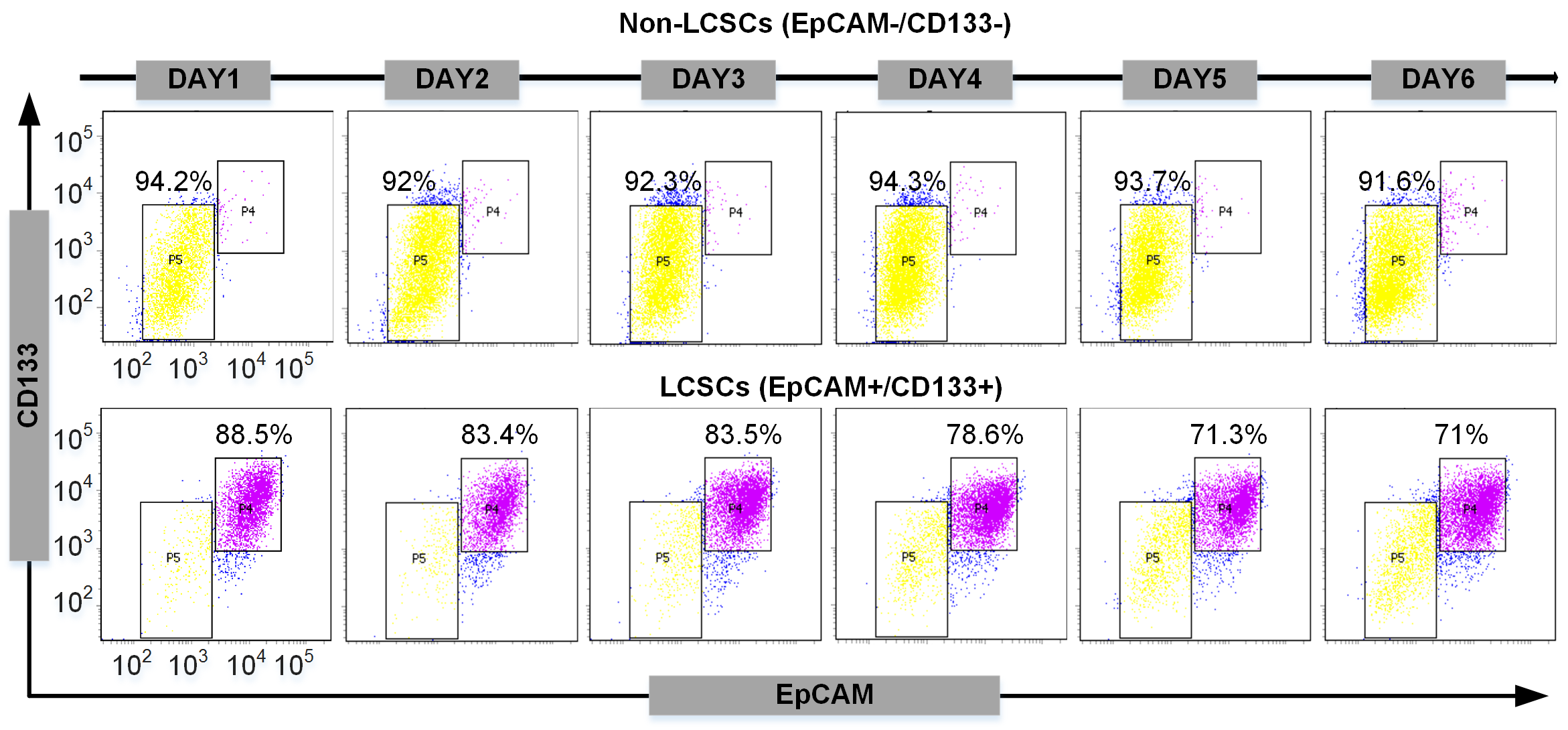

Supplement: Supplementary file 1 [file cells-09-01198-s001.zip › SupFiG1.tif]

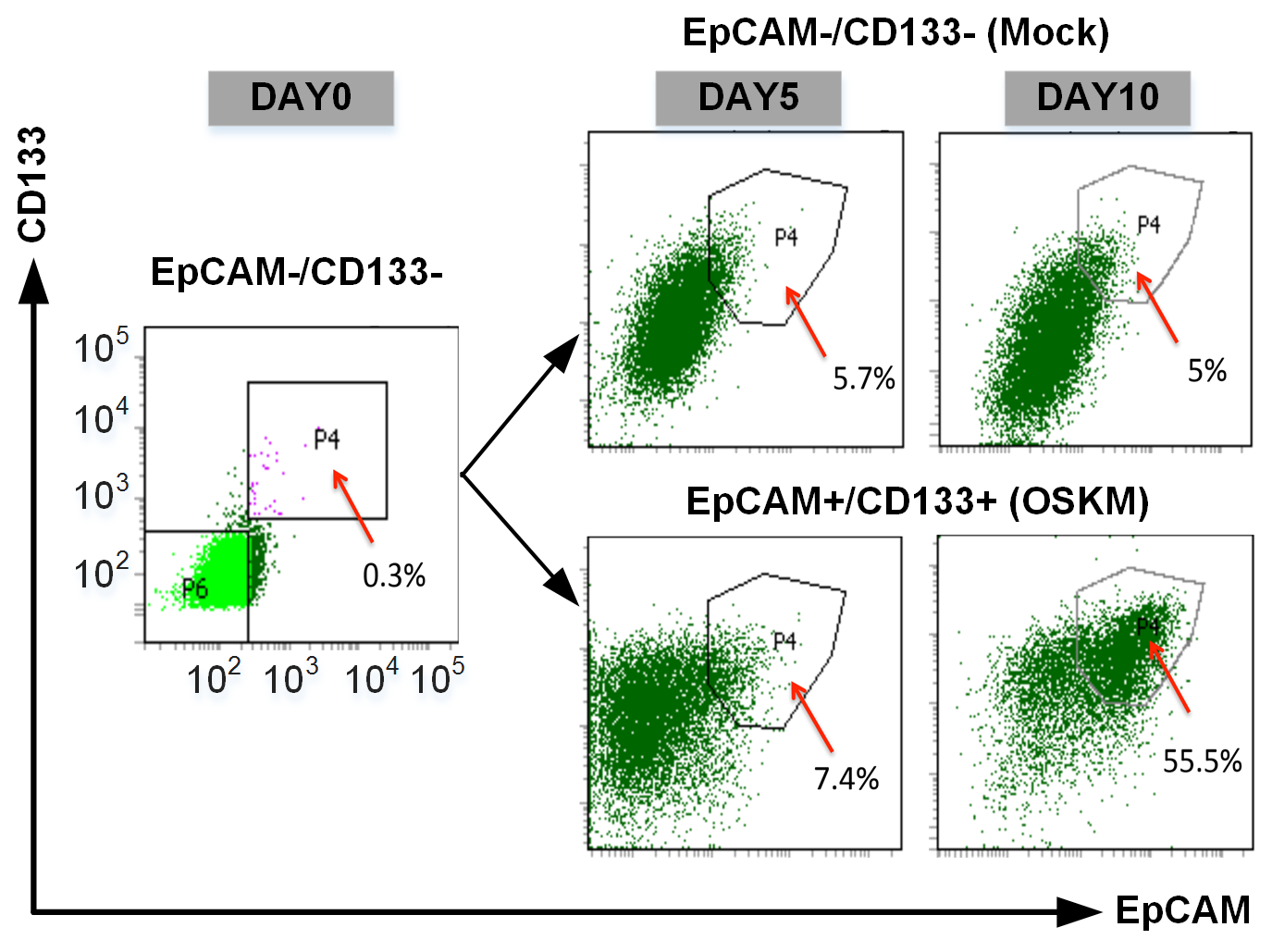

Supplement: Supplementary file 1 [file cells-09-01198-s001.zip › SupFiG2.tif]
